# Mild Cognitive Impairment Is Not “Mild” at All in Altered Activation of Episodic Memory Brain Networks: Evidence from ALE Meta-Analysis

**DOI:** 10.3389/fnagi.2016.00260

**Published:** 2016-11-07

**Authors:** Pengyun Wang, Juan Li, Hui-Jie Li, Lijuan Huo, Rui Li

**Affiliations:** ^1^Center on Aging Psychology, Key Laboratory of Mental Health, Institute of Psychology, Chinese Academy of SciencesBeijing, China; ^2^Laboratory for Functional Connectome and Development, Key Laboratory of Behavioral Science, Institute of Psychology, Chinese Academy of SciencesBeijing, China; ^3^University of Chinese Academy of SciencesBeijing, China

**Keywords:** mild cognitive impairment, episodic memory, encoding, retrieval, activation likelihood estimation

## Abstract

The present study conducted a quantitative meta-analysis aiming at assessing consensus across the functional neuroimaging studies of episodic memory in individuals with amnestic mild cognitive impairment (aMCI) and elucidating consistent activation patterns. An activation likelihood estimation (ALE) was conducted on the functional neuroimaging studies of episodic encoding and retrieval in aMCI individuals published up to March 31, 2015. Analyses covered 24 studies, which yielded 770 distinct foci. Compared to healthy controls, aMCI individuals showed statistically significant consistent activation differences in a widespread episodic memory network, not only in the bilateral medial temporal lobe and prefrontal cortex, but also in the angular gyrus, precunes, posterior cingulate cortex, and even certain more basic structures. The present ALE meta-analysis revealed that the abnormal patterns of widespread episodic memory network indicated that individuals with aMCI may not be completely “mild” in nature.

## Introduction

Mild cognitive impairment (MCI) is a state where individuals display certain form of cognitive dysfunction, but still maintain the intact ability to perform basic daily activities. MCI is generally considered as a transitional stage between normal aging and clinical dementia (Petersen, 2004). A meta-analysis reported that the annual conversion rate from MCI to dementia is approximately 5–10% (Mitchell and Shiri Feshki, 2009), which is obviously higher than the incidence rates from normal elderly to dementia (1–2% per year) (Petersen, 2004). According to Petersen (2004), the MCI individuals with memory impairment are described as amnestic MCI (aMCI) and without memory impairment as non-amnestic MCI (naMCI). Furthermore, if memory is the only impaired domain, the aMCI individuals are then classified into the aMCI-single domain; if other domains besides memory—such as language, attention/executive function, or visuospatial skills, etc.—are impaired as well, such aMCI individuals are classified into the aMCI-multiple domain. Approximately 80% of individuals with aMCI progress to Alzheimer's disease (AD) which is the most common form of dementia after a clinical follow-up of 6 years (Petersen, 2004). Thus, aMCI individuals have been receiving increasing attention.

Episodic memory is one of the earliest cognitive functions which are impaired in both early AD (Petersen et al., 1999; Perri et al., 2007) and aMCI (Bäckman et al., 2005). Impairment of episodic memory may precede dementia by as many as 10 years during when a diagnosis of aMCI may be applicable (Dannhauser et al., 2008). Typically, episodic memory is measured by tests that require knowledge of a prior episode, such as free recall, cued recall, or recognition tests (Yonelinas, 2001). Therefore, episodic memory impairment may arise from a deficiency in encoding information and/or retrieving previously stored information. And both encoding and retrieval success are associated with activation in the medial temporal lobe (MTL), prefrontal cortex (PFC), and parietal regions (Diana et al., 2007; Spaniol et al., 2009).

Task-related neuroimaging studies with positron emission tomography (PET) and functional magnetic resonance imaging (fMRI) have been increasingly conducted to examine episodic memory function in individuals with aMCI, because the future diagnostic system and successful treatment require standardized imaging inclusion criteria and isolating imaging markers which can predict the disease. Unfortunately, it is difficult to achieve consensus in activation patterns within episodic memory studies, although existing findings indicate that individuals with aMCI showed comparable fMRI test-retest reproducibility to those of healthy controls (Clément and Belleville, 2009). For instance, some studies have observed increased activation in the MTL during episodic memory encoding among aMCI individuals relative to normal controls (Dickerson et al., 2004, 2005; Kircher et al., 2007). Other studies, however, found decreased activation in the MTL during similar encoding tasks (Mandzia et al., 2009; Hanseeuw et al., 2011). Regarding the pattern of activity in neocortical areas, there is an absence of consensus in the literature of individuals with aMCI. In some studies, MTL dysfunction went with concomitant increases in PFC activity, parietal or other sites in both encoding (Heun et al., 2007; Clément and Belleville, 2012) and retrieval (Jin et al., 2012). This leads to the possibility that such changes may represent compensatory increases as a result of the MTL dysfunction. However, such findings are not universal. In other studies, individuals with aMCI showed less encoding activation in certain regions of frontal and parietal lobes (Johnson et al., 2006; Machulda et al., 2009 for retrieval). These aforementioned inconsistencies may reflect differences in participant samples, task paradigms, and methodologies across studies, which resulted in inconsistent conclusions regarding consistent brain activation patterns.

The activation likelihood estimation (ALE) is a quantitative meta-analytic procedure that has been frequently used to examine the stereotactic brain coordinates most consistently active across studies (Schwindt and Black, 2009; Browndyke et al., 2013). With an ALE analysis (Browndyke et al., 2013) evaluated inconsistent results in the episodic memory encoding literature assessing individuals with MCI and AD. However, their meta-analysis covered very few studies of individuals with MCI (8 studies as of December 31, 2009). The recently burgeoning literature assessing neural correlates of episodic memory among aMCI individuals has created an interest within this area. Additionally, it is worth noting that despite the large number of neuroimaging studies concerning episodic memory retrieval in aMCI, to our knowledge, there is no meta-analyses addressing consensus activation patterns within this participant group. Accordingly, by using a quantitative ALE meta-analysis, the goal of the present study was to establish consistently robust patterns during both memory encoding and retrieval across several studies assessing episodic memory in MCI individuals. Considering the difference of dysfunction between individuals with aMCI and naMCI, and the seldom studies of individuals with naMCI, only studies with aMCI participants are contained in the present Meta-analysis, including both single domain and multiple domain aMCI.

## Methods

### Literature collection and criteria

An initially broad and thorough literature search was implemented on PubMed, Web of Knowledge, and EBSCO (PsyINFO, PsycARTICLES, PsycCRITIQUES, PsycEXTRA, and PsycTESTS) searchesto collect functional imaging studies assessing individuals with aMCI using the following key words: (functional Magnetic Resonance Imaging OR fMRI OR positron emission tomography OR PET) AND (mild cognitive impairment OR MCI) AND (memory OR recognition OR recall). These searches were confined to articles published between January 1, 1990 (which was early enough for searching the studies on MCI) and March 31, 2015, which yielded 2059 unique research or review articles.

Within these studies, only those that met the following criteria were examined and taken into consideration: (1) performing the diagnosis of aMCI according to Petersen et al. (2001); Petersen (2004); or Winblad et al. (2004) was; (2) reporting PET or fMRI results of episodic encoding and/or retrieval paradigms compared to baseline task(s); (3) describing results of independent groups (MCIs and matched controls) or between-group comparisons based on a whole-brain analysis; and (4) using standard stereotactic coordinates to list peaks of significant activation (Talairach and Tournoux, 1988) or Montreal Neurologic Institute (MNI) space.

According to the criteria mentioned above, all articles were reviewed by two independent raters (Pengyun Wang and Lijuan Huo). After applying the first two inclusion criteria, 2018 unrelated articles were excluded and 41 related articles were left. These articles were then subject to further consideration on the basis of inclusion Criteria three and four. Thirteen studies did not meet the criteria three, for their results based on a priori cortical ROIs, and either did not conduct whole-brain voxel-wise analyses, simply reporting differences in activation only in certain specific areas (Dickerson et al., 2004, 2005; Johnson et al., 2006, 2008; Sandstrom et al., 2006; Mevel et al., 2007; Xu et al., 2007; Chao et al., 2009; Yassa et al., 2010; Miettinen et al., 2011; Putcha et al., 2011; Trivedi et al., 2011; Bakker et al., 2015). Such an analysis may partially emphasize some regions and ignore others. Two studies were ruled out, because their stereotactic results were not reported (Dhanjal et al., 2013; Dhanjal and Wise, 2014). Another two studies were excluded because they did not reveal any differences in activation between individual with aMCI and normal controls (Parra et al., 2013; Nicholas et al., 2014). As emphasized above, only individuals with aMCI (including both aMCI-single and aMCI-multiple domain) were included in the current meta analysis. In the study of Machulda et al. (2009), for example, participants of naMCI were excluded, but the ones of aMCI were included.

A final set of 24 studies (publication dates range from 2006 to 2013) was included in the current analysis, yielding 770 distinct foci for the ALE meta-analysis (Table 1).

**Table 1:** **Characteristics of studies included in the meta-analysis of fMRI studies of mild cognitive impairment**.

**Articles**	**Subjects**	**Age (SD)**	**Education (SD)**	**MMSE (SD)**	**Modality**	**Task**	**Task paradigm**	**Contrast condition**	**Group Contrasts and Number of foci**
Celone et al., 2006	27 MCI 15 controls	77.3 (6.1) 75.5 (6.0)	16.3 (3.1) 16.5 (2.1)	29.0 (1.0) 29.5 (0.5)	fMRI	Encoding	Face-name associative learning	Encoding component (ica data)^*^	NC 2 MCI 2 MCI > NC 4
Clément and Belleville, 2010	26 MCI 14 controls	67.9 (8.5) 67.2 (6.8)	14.4 (3.9) 14.6 (3.8)	27.7 (1.6) 29.3 (1.1)	fMRI	Encoding	Semantically related/unrelated word-pair learning	Encoding word-pairs > visual fixation	NC 12 MCI 28 MCI < NC 2 MCI > NC 6
Clément et al., 2010	12 MCI 10 controls	67.8 (7.5) 71.7 (7.6)	13.3 (4.0) 12.5 (2.7)	27.8 (1.6) 29.1 (0.7)	fMRI	Encoding Retrieval	Words learning and recognition	Encoding/ retrieval vs. Rest	MCI < NC 6 MCI > NC 1 MCI < NC 1 MCI > C 1
Clément and Belleville, 2012	26 MCI 14 controls	67.9 (8.5) 67.2 (6.8)	14.5 (3.9) 14.6 (3.8)	27.7 (1.6) 29.3 (1.1)	fMRI	Retrieval	Item and associative word-pair recognition	Recognition (old/new or intact/rearranged) > visual fixation	NC 10 MCI 37 MCI < NC 1 MCI > NC 10
Dannhauser et al., 2008	10 MCI 10 controls aMCI	72.0 (7.7) 68.0 (13.5)	10.3 (1.8) 10.1 (1.4)	24.5 (1.5) 28.3 (1.6)	fMRI	Encoding	Visual Verbal encoding	Encode vs. Visual control condition	NC 2 MCI 3 MCI < NC 1
de Rover et al., 2011	15 MCI 16 controls	69.3 (4.2) 65.7 (5.6)	–	25.9 (1.3) 28.2 (1.4)	fMRI	Encoding Retrieval	Object-location associations memory	Encoding/retrieval vs. Visual control condition	NC 9 MCI 9 NC 2 MCI 2
Giovanello et al., 2012	12 MCI 12 controls	75.2 (4.3) 72.6 (5.9)	16.3 (2.9) 15.6 (3.1)	27.8 (1.7) 29.5 (0.9)	fMRI	Retrieval	Item and associative word-pair recognition	Relational memory vs. Item memory	NC 3 MCI 4 MCI < NC 4 MCI > NC 2
Gronholm et al., 2007	10 MCI 10 controls	68.6 (8.6) 65.5 (6.9)	11.2 (3.3) 11.3 (3.9)	27.3 (1.5) 29.0 (0.7)	PET	Retrieval	Non-living objects memory	Familiar non-living objects vs. Visual noise patterns	NC 3 MCI 4
Hämäläinen et al., 2007	14 MCI 21 controls	72.4 (7.3) 71.2 (4.9)	8.1 (2.6) 7.9 (2.9)	25.6 (3.1) 27.7 (2.0)	fMRI	Encoding	word-picture pairs learning	Encoding vs. Visual fixation baseline	NC 28 MCI < NC 1 MCI > NC 13
Hampstead et al., 2011	18 MCI 16 controls	71.2 (8.5) 72.1 (7.3)	17.1 (2.1) 16.1 (2.7)	26.7 (2.3) 27.8 (2.0)	fMRI	Encoding	Object-location associations memory	Successfully encoded novel vs. Repeated contrast.	NC 93 MCI 60 MCI < NC 100
Hanseeuw et al., 2011	16 MCI 15 controls	72.6 (7.9) 69.4 (4.8)	13.5 (2.7) 14.9 (2.4)	27.3 (1.6) 28.7 (1.5)	fMRI	Encoding	Cue-item association learning	Successful associative encoding vs. Visual fixation baseline	MCI < NC 5
Heun et al., 2007	20 MCI 28 controls	69.7 (7.1) 67.5 (5.4)	–	26.6 (1.5) 28.9 (1.1)	fMRI	Retrieval	Words recognition	Word retrieval vs. visual fixation baselin	NC 1 MCI 3 MCI > NC 3
Jin et al., 2012	8 MCI 8 controls	60.9 (3.2) 60.6 (8.3)	16.9 (1.9) 16.9 (2.1)	28.1 (1.1) 29.6 (0.5)	fMRI	Encoding Retrieval	Pictures of scene learning, faces and occupations pairs learning, objects and locations learning	Encoding/retrieval vs. Visual control condition	MCI < NC 2 MCI > NC 1 MCI < NC 8 MCI > NC 4
Kircher et al., 2007	21 MCI 29 controls	69.7 (7.0) 67.8 (5.4)		26.6 (1.4) 28.8 (1.2)	fMRI	Encoding	Visual words learning	Hit vs. Misses	NC 4 MCI 5 MCI > NC 4
Lenzi et al., 2011	15 MCI 14 controls	72.5(58–85) 64.3(50–81)	10.3(5–17) 13.6(5–17)	25.2 (23–27) 28.6(25–30)	fMRI	Retrieval	Sentences (sound) recognition	Recognition vs. Tones (baseline)	NC 5 MCI 5 MCI > NC 1
Li et al., 2013	34 aMCI 25 controls	64.38 62.52 (5.41)	11.11 Abnormal data	26.00 28.64 (1.44)	fMRI	Encoding	Natural and artificial picture learning	Encoding vs. visual fixation baseline	MCI < NC 9
Machulda et al., 2009	19a MCI 29 controls	76.6 (6.8) 73.0 (7.0)	14.9 (3.4) 14.1 (2.4)		fMRI	Encoding Retrieval	Pictures of Scene encoding and recognition	Encoding/retrieval block vs. Baseline task block	MCI < NC 7 MCI < NC 4
Mandzia et al., 2009	14 MCI 14 controls	68.6 (7.4) 72.2 (6.4)	13.4 (2.8) 15.4 (2.8)	27.7 (1.1) 28.6 (1.1)	fMRI	Encoding Retrieval	Pictures of objects and animals encoding and recognition	Encoding/retrieval block vs. Baseline task block	MCI < NC 23 MCI < NC 13 MCI > NC 4
Moulin et al., 2007	31 MCI 29 controls	67.1 (6.7) 65.9 (5.5)	–	27.6(1.1) –	PET	Encoding Retrieval	Word-pair learning	Encoding/retrieval block vs. Baseline task block (visual words)	NC 3 MCI 2 NC 2 MCI 3
Petrella et al., 2006	20 MCI 20 controls	75.0 (7.6) 71.2 (4.5)	15.0 (2.2) 15.9 (2.9)	26.7 (1.5) 28.4 (1.4)	fMRI	Encoding Retrieval	Face-name associative learning	Novel pairs vs. Repeated pairs	MCI < NC 5 MCI < NC 6 MCI > NC 2
Petrella et al., 2007	34 MCI 28 controls	74.5 (8.6) 72.0 (5.0)	15.1 (2.5) 16.3 (2.8)	26.7 (1.9) 28.3 (1.4)	fMRI	Encoding	Face-name associative learning	Novel encoding vs. Repeated encoding	NC 15 MCI 15 MCI < NC 8 MCI > NC 10
Ries et al., 2006	14 MCI 14 controls	73.7 (6.9) 72.5 (5.7)	16.2 (2.7) 17.3 (2.9)	28.6 (1.5) 29.4 (0.8)	fMRI	Retrieval	Visual item recognition	Old vs. New	NC 7 MCI 3
Trivedi et al., 2008	16 MCI 23 controls	73.1 (5.5) 77.0 (8.4)	14.9 (3.3) 16.2 (3.0)	26.3 (2.3) 28.8 (1.2)	fMRI	Encoding Retrieval	Visual objects encoding and recognition	Encoding novel vs. Repeated word “push” Hits vs. Misses	NC 12 MCI 12 MCI < NC 8 MCI > NC 1 MCI < NC 17 MCI > NC 1
van der Meulen et al., 2012	13 MCI 15 controls	69.2 (8.2) 68.1 (7.2)	13.0 (2.3) 14.3 (2.6)	26.7 (2.3) 29.5 (0.8)	fMRI	Encoding Retrieval	Picture pairs memory	Encoding/retrieval block vs. Resting baseline	NC 14 MCI 9 MCI < NC 7 NC 12 MCI 11 MCI < NC 8

*MMSE, mini mental status examination; SD, standard deviation*.

* *ICA, independent component analysis*.

### ALE analysis

The software GingerALE 2.3 (Turkeltaub et al., 2002; Eickhoff et al., 2009) was used to conduct these ALE meta-analyses. To allow for direct comparisons of spatial brain coordinates across studies, relevant foci in the included studies were converted from the Talairach and Tournoux (1988) atlas into MNI space, using the Lancaster transform (Lancaster et al., 2007) implemented in the GingerALE software (www.brainmap.org/ale/). The activation foci were then modeled as the center of a 10-mm^3^ full width-at-half-maximum Gaussian sphere. The ALE statistical test represents the probability that a voxel contains at least one of the activation foci. The GingerALE software compares the resultant ALE maps to the averaged map from 5000 permutations of an identical number of foci placed randomly throughout the brain, controlling the false discovery rate alpha cut-off of 0.05 during the multiple comparisons (Laird et al., 2005). A cluster threshold with a minimum volume of 100 mm^3^ was applied. ALE analysis clusters were required to have contributing spatial coordinates from a minimum of two independent studies shown in Table 1. The results of these ALE analyses were viewed using the MRIcroN (http://www.nitrc.org/projects/mricron/). The template was “Colin27_T1_seg_MNI” (http://www.brainmap.org/ale/).

Eight separate ALE analyses were performed in two ways. First, analyses were run for individuals with aMCI and healthy controls separately, by computing the activated foci during encoding and retrieval separately. Then, the ALE analyzed the attenuated (healthy controls > individuals with aMCI) and hyperactivated (individuals with aMCI > healthy controls) brain foci during encoding and retrieval separately.

## Results

**Figure 1:**
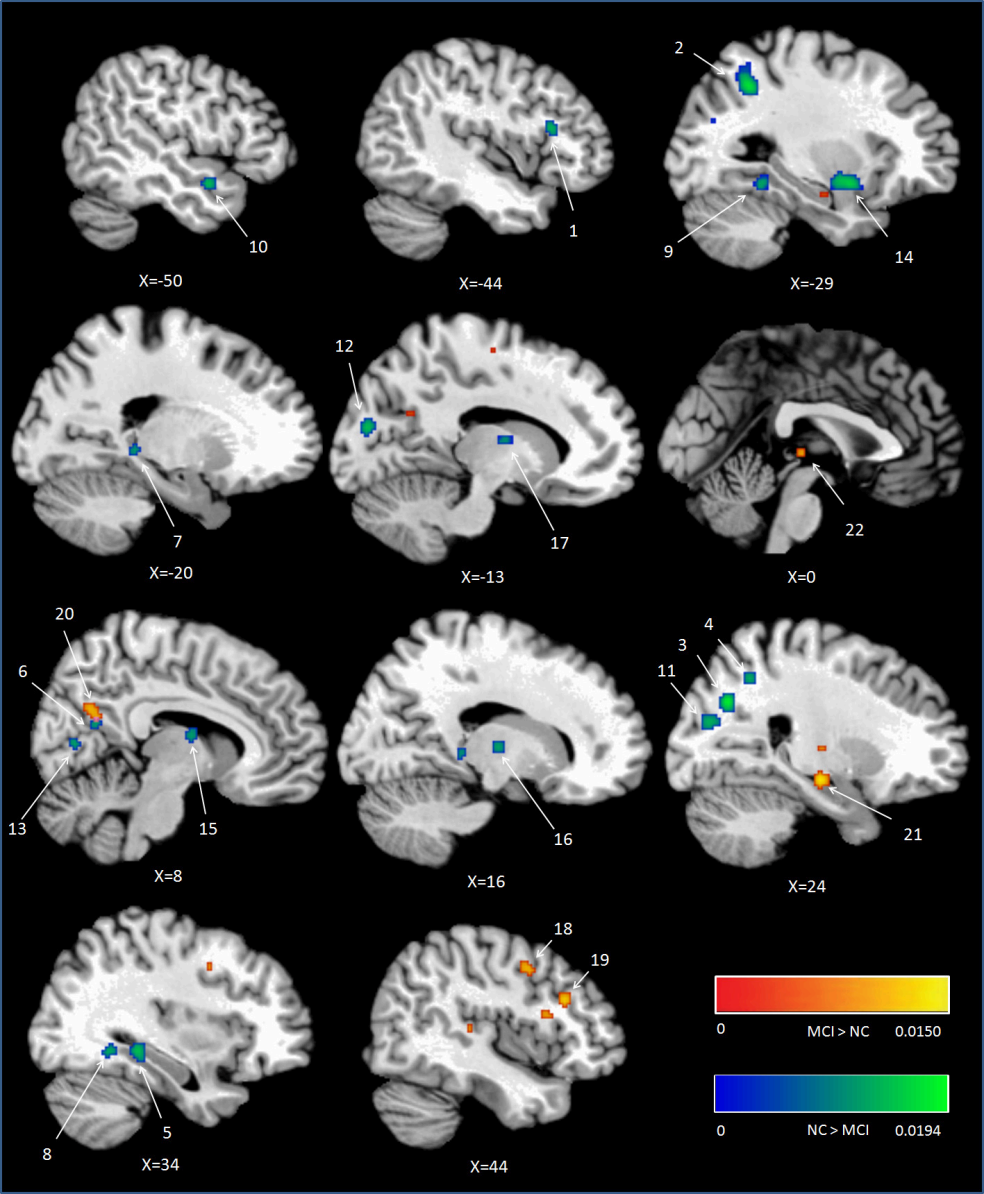
**Cluster results of ALE comparison analysis between individuals with aMCI and healthy controls across encoding studies**. MCI, mild cognitive impairment; NC, normal control.

**Figure 2:**
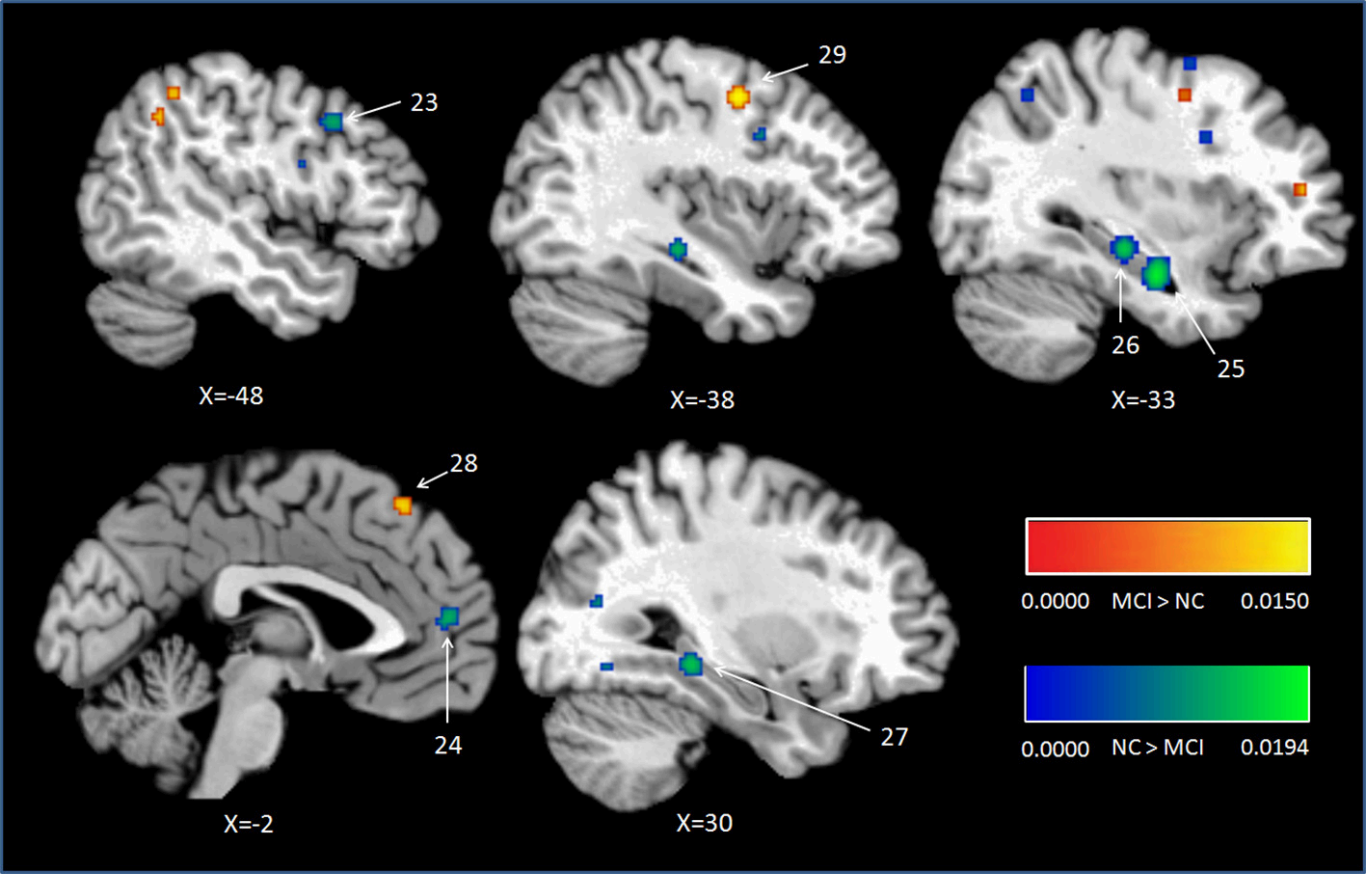
**Cluster results of ALE comparison analysis between individuals with aMCI and controls across retrieval studies**. MCI, mild cognitive impairment; NC, normal control.

Peak MNI coordinates, Brodmann areas (BA), and cluster sizes of significant ALE regions are summarized in Table 2 (group difference between aMCI individuals and healthy controls during encoding and retrieval respectively) and Supplementary Table 1 (within group activation of individuals with aMCI and healthy controls during encoding and retrieval respectively). The ALE values showed in these two tables are the maximum activation likelihood estimates for individual statistically significant clusters. Thresholded ALE spatial maps for regions of general difference between aMCI individuals and healthy controls were presented in Figure 1 for encoding and Figure 2 for retrieval.

**Table 2:** **Results of ALE analyses for group comparison**.

**Cluster no**.	** Region (left/right, Brodmann area)**	***X***	***Y***	***Z***	**Cluster size (mm^3^)**	**ALE (× 10^−2^)**
	**Encoding**					
	**MCI** < **NC**					
	**Frontal lobe**					
1	Middle frontal gyrus (L, 46)	−44	22	16	176	1.01
	**Parietal lobe**					
2	Angular gyrus (L, 39)	−30	−54	42	896	1.83
3	Precuneus (R, 31)	22	−68	30	520	1.95
4	Precuneus (R, 7)	24	−56	44	168	1.34
	**Limbic lobe**					
5	Hippocampus (R)	32	−36	−10	680	1.60
6	Posterior cingulate (R, 23)	6	−60	18	264	1.54
7	Parahippocampal gyrus (L, 27)	−20	−36	−2	112	1.19
	**Temporal lobe**					
8	Fusiform gyrus (R, 37)	38	−54	−10	392	1.47
9	Fusiform gyrus (L, 37)	−28	−46	−14	320	1.36
10	Superior temporal gyrus (L, 38)	−50	2	−18	240	1.43
	**Occipital lobe**					
11	Cuneus (R, 17)	24	−78	20	400	1.33
12	Cuneus (L, 17)	−14	−86	16	392	1.47
13	Lingual gyrus (R, 18)	8	−72	6	112	1.23
	**Deep gray structures**					
14	Lentiform nucleus, putamen (L)	−28	4	−14	752	1.60
15	Thalamus, anterior nucleus (R)	8	−6	10	168	1.20
16	Thalamus, ventral lateral nucleus (R)	16	−14	4	136	1.28
17	Thalamus, ventral lateral nucleus (L)	−12	−8	10	120	1.14
	**MCI** > **NC**					
	**Frontal lobe**					
18	Precentral gyrus (R, 6)	42	2	42	472	1.12
19	Middle frontal gyrus (R, 9)	46	24	24	424	1.50
	**Parietal lobe**					
20	Precuneus (R, 31)	8	−64	26	248	1.04
		10	−60	22		
	**Deep gray structures**					
21	Lateral globus pallidus (R)	26	−16	−14	376	1.44
22	Thalamus (L)	0	−22	0	144	0.98
	**Retrieval**					
	**MCI** < **NC**					
	**Frontal lobe**					
23	Middle frontal gyrus (L, 9)	−48	14	36	488	1.01
		−42	8	38		0.88
24	Medial frontal gyrus (L, 9)	−2	52	10	312	1.05
	**Limbic lobe**					
25	Hippocampus (L)	−32	−12	−22	816	1.54
26	Hippocampus (L)	−34	−24	−12	472	1.38
27	Hippocampus (R)	30	−34	−10	400	1.15
	**MCI** > **NC**					
	**Frontal lobe**					
28	Superior frontal gyrus (L, 8)	0	36	52	344	1.06
29	Middle frontal gyrus (L, 6)	−38	0	48	256	1.03

*ALE, activation likelihood estimation. Coordinates in stereotactic space of MNI*.

### Encoding

#### NC

Eleven of the twenty-four fMRI episodic memory studies reported activated foci in healthy controls alone. The current analysis included a total of 194 foci and 216 healthy controls. During encoding, NC demonstrated elevated activation likelihood in a range of prefrontal, parietal, limbic, and some other cortical sites. Within the frontal lobe, elevated values were showed in the bilateral DL-PFC (L, BA9; R, BA46), regions of the dorsal lateral surface in the right precentral gyrus (BA6), left medial frontal gyrus (BA6), and left superior frontal gyrus (BA6). Increased activation likelihood in the limbic lobe was observed in the bilateral parahippocampal gyrus (R, L, BA27 and L, BA36). The peaks of these clusters were located in the bilateral entorhinal cortex and the left perirhinal cortex. Increased activation likelihood in the parietal lobe was seen in the bilateral precuneus (BA7 and BA19). Additional areas of high likelihood were found in the left fusiform gyrus (BA37), right medial globus pallidus, left sub-lobar thalamus, left thalamus, left insular cortex (BA13), left amygdala, right lingual gyrus (BA18), right cuneus (BA17), right middle occipital gyrus (BA19), and two clusters in bilateral cerebellum. See Supplementary Table 1.

#### MCI

Eleven of the twenty-four fMRI episodic memory studies reported activated foci in aMCI individuals alone. The analysis included a total of 177 foci and 225 individuals with aMCI. During encoding, individuals with aMCI showed large areas of prefrontal activation, with multiple clusters in the right DL-PFC (BA9), regions of the dorsal lateral surface in bilateral precentral gyrus (BA6), and left medial frontal gyrus (BA6). Increased activation likelihood in the limbic lobe was observed in the bilateral entorhinal (BA27, 28) and perirhinal cortex (BA36), while no hippocampal peaks were seen. Parietal involvement was limited to the area of the right superior parietal lobule (BA7) and bilateral precuneus (BA7). Additional peaks were found bilaterally in the left sub-lobar thalamus pulvinar, superior temporal gyrus (BA22, 38), left fusiform gyrus (BA37), bilateral lingual gyrus (BA18), bilateral middle occipital gyrus (L, BA18; R, BA19), right cuneus (BA17), and cerebellum. See Supplementary Table 1.

#### MCI < controls

Fourteen studies with 184 foci provided information about attenuated brain activation of individuals with aMCI when performing the episodic encoding processing relative to healthy controls. The ALE analysis indicated that individuals with aMCI showed consistently lower activation likelihood in a range of sites in frontal, parietal and limbic lobe, including the left DLPFC (Cluster No. 1 in Tabel 2), left angular gyrus (BA39), right precuneus (BA31/7), right hippocampus, right posterior cingulate (BA23), and left parahippocampal gyrus (BA27). Three temporal peaks referred to bilateral fusiform gyrus (BA37) and left superior temporal gyrus (BA38). Other peaks were observed in the bilateral cuneus (BA17), lingual gyrus (BA18) and a few deep gray structures in the left lentiform nucleus and bilateral thalamus. See Table 2 and Figure 1.

#### MCI > controls

Eight studies reported 40 brain foci with higher activation in aMCI individuals in the current ALE analysis. The results indicated that individuals with aMCI also demonstrated greater activation likelihood compared to healthy controls in several regions. Two prefrontal involved the right DLPFC (BA9) and a region of the dorsal surface in the right precentral gyrus (BA6). Other peaks were observed in the right precuneus (BA31), the right lateral globus pallidus, and left thalamus. See Table 2 and Figure 1.

### Retrieval

#### NC

Nine fMRI episodic retrieval studies reported activated foci in healthy controls alone. The analysis contained a total of 45 foci and 152 healthy controls. At retrieval, increased activation likelihood in NC were seen in the medial part of right superior frontal gyrus (BA6), left precuneus (BA7), and left middle temporal gyrus (BA39). Additional peaks were found in the right cuneus (BA18), right extra-nuclear (BA7), and part of left cerebellum. See Supplementary Table 1.

#### MCI

Nine studies reported activated foci in individuals with aMCI alone. The ALE analysis included a total of 72 foci and 156 individuals with aMCI. At retrieval, increased activation likelihood in individuals with aMCI were oberserved in the medial part of right superior frontal gyrus (BA6) and bilateral precuneus (BA7). Additional peaks were found in the right lingual gyrus (BA18), right claustrum, and part of right cerebellum. See Supplementary Table 1.

#### MCI < controls

Nine studies with 63 foci provided information aboutattenuated brain activation of individuals with aMCI when performing the episodic retrieval processing compared to healthy controls. The ALE analysis indicated that individuals with aMCI demonstrated lower activation likelihood relative to controls within left areas of the DLPFC (Cluster No. 23 in Table 2), mPFC (Cluster No. 24 in Table 2), and the bilateral hippocampus. See Table 2 and Figure 2.

#### MCI > controls

Nine studies reported 28 brain foci with higher activation in individuals with aMCI in the current ALE analysis. Compared to healthy controls, aMCI individuals demonstrated greater activation likelihood only in two prefrontal areas, the left middle frontal gyrus (BA6) and left superior frontal gyrus (BA8). See Table 2 and Figure 2.

## Discussion

For aMCI and NC respectively, the results of the present study demonstrated that both of the two groups showed elevated activation likelihood during encoding, involving DLPFC, dorsal frontal cortex, precuneus, parahippocampal gyrus, fusiform gyrus, lingual gyrus, cuneus and certain more basic structures. During retrieval, the consistent activations in both aMCI and NC were observed in the dorsal frontal cortex, the precuneus, the cuneus and some regions in sub-lobar and cerebellum.

What was mainly concerned in the present meta-analysis, however, was the group differences between aMCI individuals and the healthy controls. Compared to healthy controls, individuals with aMCI showed statistically significant consistent activation differences in a widespread episodic memory network, not only in the bilateral medial temporal lobe and prefrontal cortex, but also in the angular gyrus, precunes, posterior cingulate cortex, and even some more basic structures such as the thalamus, fusiform gyrus, and cuneus.

### MTL structures

The results on episodic encoding in the present meta-analysis were inconsistent with the previous founding by Browndyke et al. (2013). Specifically, in the present study, individuals with aMCI showed reliably lower activation likelihood in the right hippocampus and left parahippocampal gyrus compared to normal controls. These regions belong to MTL, which is crucial to episodic encoding. In line with the findings of meta-analysis in AD patients (Schwindt and Black, 2009), the result of present study indicated that the lower activation in MTL structures during encoding processing led to memory impairment. In contrast, Browndyke et al. (2013) found that aMCI individuals showed higher activation likelihood within a region near the right perirhinal cortex (BA35) during memory encoding. They proposed that this may reflect an increase or overreliance on familiarity-based processing during episodic encoding in MCI, not necessarily being able to benefit successful memory retrieval. The present meta-analysis differed from that of Browndyke et al. (2013) mainly in the literature included in the ALE analysis. In respect of the MTL region, comparing with Browndyke et al.'s research, the present study included more studies which reported lower activation foci during encoding. Specifically, in the present study, five studies (Celone et al., 2006; Hämäläinen et al., 2007; Kircher et al., 2007; Trivedi et al., 2008; Clément and Belleville, 2010) reported seven elevated activation foci in individuals with aMCI in the MTL structure during encoding. Browndyke et al. (2013), however, included four of them except one (Clément and Belleville, 2010). With respect to the lower activation foci in aMCI in the MTL structure, six studies (Trivedi et al., 2008; Mandzia et al., 2009; Hampstead et al., 2011; Hanseeuw et al., 2011; Jin et al., 2012; van der Meulen et al., 2012) were analyzed in the present study providing nine foci. Browndyke et al. (2013), however, included only three studies (Johnson et al., 2006, 2008; Trivedi et al., 2008) with five foci. The first two were excluded in the present study because of their ROI analysis method. Moreover, these two studies did not lead to any difference in the MTL region between the present study and the meta-analysis by Browndyke et al. (2013), because neither of them found any areas where individuals with MCI had elevated activation during encoding. In short, far more lower activation foci, which were found in the recently years, were contained in the present meta-analysis. Therefore, the different results may be due to the insufficient number of studies examined in the previous meta-analysis (Browndyke et al., 2013). The results of the present study are relatively more reliable.

Consistent deficits in activation within MTL structures were observed among individuals with aMCI during retrieval. The peaks of the clusters were located in bilateral hippocampus as showed in Figure 2, the clusters with lower activation likelihood involved large regions in bilateral hippocampus of aMCI individuals. It is well established that hippocampus play a critical role in episodic memory retrieval (Diana et al., 2007; Spaniol et al., 2009). The dysfunction of hippocampus in individuals with aMCI impaired the access of information which has been stored previously.

In short, areas in MTL structures showed lower activation among individuals with aMCI compared to healthy controls during both encoding and retrieval processing. It is reported that the earliest brain changes in MCI, as measured by volume loss, occur in the hippocampus and entorhinal cortex of the MTL (Masdeu et al., 2005). Considering the crucial function of MTL in episodic memory (Eichenbaum et al., 2007), these findings suggest that volumetric and functional reductions in MTL have a significant impact on the episodic encoding and retrieval impairments observed in MCI.

### Frontal regions

The results of current meta-analysis indicated that individuals with aMCI showed different patterns in encoding and retrieval phases in frontal regions. In encoding phase, healthy controls demonstrated greater activation likelihood in the left DLPFC (BA46) relative to individuals with aMCI. In the meanwhile, individuals with aMCI showed elevated likelihood in the right dorsal frontal cortex (BA 6) and the right DLPFC (BA 9). In retrieval phase, lower activation likelihood in the left DLPFC and mPFC, but greater likelihood in the left dorsal frontal cortex (BA 6 and 8) were found in individuals with aMCI compared to controls.

In the present ALE analysis, the greater activity of right DLPFC during encoding in the aMCI individuals is possible to be a compensation for their lower activity of left DLPFC. Converging evidence indicates that the DLPFC specifically contributes to successful memory formation through its role in building relation among items (Dolan and Fletcher, 1997; Murray and Ranganath, 2007; Blumenfeld et al., 2011). In the present meta-analysis, the two foci contributing to the cluster of left DLPFC (lower likelihood in individuals with aMCI) during encoding were reported by two papers (Petrella et al., 2006; Dannhauser et al., 2008), while the contributors to the cluster in right DLPFC (greater likelihood in individuals with aMCI) by another study (Clément and Belleville, 2010). In the last study, the participants with aMCI were divided into two groups of different levels of cognitive impairment (aMCI higher-cognition and aMCI lower-cognition) according to a split-median of their scores on the Mattis Dementia Rating Scale (MDRS), which is an abbreviated neuropsychological scale that covers a wide range of cognitive functions. The aMCI higher-cognition participants showed more activity in these two areas in right DLPFC relative to healthy controls. As argued by the authors, the aMCI higher-cognition participants achieved comparable performances of healthy controls. This suggested that their additional right DLPFC activations reflected compensatory mechanisms. In contrast, the aMCI lower-cognition participants in this study exhibited lower activity in right DLPFC, and they did not exhibited additional activations in right or left DLPFC when comparing to the controls. In short, the result of the present ALE analysis argues that the greater activity of right DLPFC in the aMCI individuals is possible to be a compensation for their lower activity of left DLPFC and the MTL region during encoding episodic information, especially for the aMCI with higher-cognition. This explanation is in line with the degeneration models, which propose that there is a trade-off between the accumulation of lesions and the ability for the neural system to exhibit compensation (Friston and Price, 2003; Cabeza and Dennis, 2012). Furthermore, this change of activity showed in the dominant (left) DLPFC and compensatory (right) DLPFC was also in agreement with the phenomenon of increased bilaterality in frontal areas, which is clearly established in normal aging [see review of Craik and Rose, 2012 and the ‘scaffolding theory of aging and cognition’ (STAC) Park and Reuter-Lorenz, 2009].

The role of DLPFC has also been demonstrated to provide top–down input to the medial temporal lobe in support of retrieval (Tomita et al., 1999), such as monitoring the outcome of retrieval attempts (Fletcher et al., 1998; Fletcher and Henson, 2001). Recently some research suggested that intentional retrieval was associated with increased activation in DLPFC (Kompus et al., 2011). Moreover, attempts to stop memory retrieval are also associated with greater activation of lateral prefrontal cortex than attempts to retrieve memories (for review, see Anderson and Huddleston, 2012). A DLPFC-cingulate-parietal-hippocampal network has been demonstrated to exhibit strongly correlated activity during retrieval suppression. Individuals who were able to suppress memory retrieval exhibited tighter coupling between the key nodes in this network than individuals who were not (Paz-Alonso et al., 2013). In the present study, the lower activation likelihood in left DLPFC during memory retrieval indicated the deficit of aMCI individuals in top–down memory retrieval control which is related to their insufficient performance of episodic memory.

However, we also observed additional activations both during encoding and retrieval in the dorsal frontal cortex that was usually not reported as being involved in verbal episodic tasks. Those regions are localized in premotor cortex and supplementary motor cortex regions [i.e., the right precentral gyrus (BA6), superior frontal gyrus (BA8) and middle frontal gyrus (L, 6)]. These new activations may represent the recruitment of additional compensatory networks for the disrupted function in DLPFC. The additional activations in the superior frontal gyrus (BA8) are more possible to agree with this hypothesis. It has been found that insufficient engagement of the superior frontal gyrus (BA8) may allow goal-irrelevant information access to working memory and to be encoded into long-term memory (Minamoto et al., 2012). Considering the dysfunctional inhibition of distracting information in AD (Baddeley et al., 2001; Amieva et al., 2004), individuals with aMCI may need more effort to regulate this form of attentional control. Alternatively, as suggested by Jin et al., the elevated activation in precentral gyrus and superior motor area in individuals with aMCI may be caused by the active control state which is not memory relevant (Jin et al., 2012). However, considering the memory task procedures used in the included studies of current meta-analysis, participants performed almost the equal active control effort in both memory processing and baseline task. Therefore, the differences between active control efforts had been counteracted by the baseline contrasting. The compensatory hypothesis may explain results of the present meta-analysis more reasonably.

Individuals with aMCI also showed lower activation likelihood in left mPFC during retrieval, which is a key region in memory network, especially in memory retrieval and consolidation (Preston and Eichenbaum, 2013). Complementary studies support the idea that the mPFC acquires representations of behavioral contexts to control memory retrieval. The interactions between the mPFC and hippocampus may support the ability to create contextual representations, and use these representations to retrieve the memories that conform to a given context (for review, see Preston and Eichenbaum, 2013). The deactivation of mPFC in aMCI individuals can be one of the most critical reasons which lead to their retrieval deficits.

In short, the current study exhibited aMCI's deficits in the left DLPFC during both encoding and retrieval, revealing greater activation in the right DLPFC and right dorsal frontal cortex during encoding, and the left dorsal frontal cortex during retrieval. These findings are different from the pattern found in AD patients (Schwindt and Black, 2009), which presented greater activity in the DL-PFC and VL-PFC but less activity in the anterior PFC regions and dorsal frontal cortex during both encoding and retrieval. Given that the increased frontal cortex activity during episodic memory is considered as compensation for the MTL dysfunction (Grady et al., 2005), these results suggest that individuals with aMCI are quite likely touse a distinctive compensatory network in frontal cortex during episodic memory relative to AD patients.

### Parietal region

As part of the medial posterior parietal cortex, the precuneus, especially the dorsal subregions of precuneus, has been acknowledged playing a central role in a wide spectrum of highly integrated tasks, including visuo-spatial imagery, episodic memory retrieval (Lundstrom et al., 2003, 2005; Dörfel et al., 2009) and self-processing operations (see Cavanna and Trimble, 2006; Cavanna, 2007 for review). Although the contribution of precuneus to successful encoding has received relatively little attention, it was still found that precuneus is involved in allocentric encoding of spatial locations (Frings et al., 2006). Furthermore, the ventral subregion of precuneus showed greater activity during resting as compared to responding to an external task (Fransson and Marrelec, 2008), and is wildly accepted as part of the default mode network (Zhang and Li, 2012). Some studies argued that the ventral subregion of precuneus (next to the posterior cingulate cortex) deactivated during successful encoding processes (Daselaar et al., 2004; Vannini et al., 2011). A recent study found that this deactivation reversed to higher activation in preclinical stage of AD compared to controls when performing a visual encoding memory task. In addition, there was a tendency negative correlation between the activations of this region and the task performance (Rami et al., 2012). In the present study, lower activations in the dorsal precuneus (region 3 and 4 in Figure 1) were observed but elevated activations in the ventral precuneus (region 20 in Figure 1) in aMCI individuals compared to controls during encoding. According to the findings of previous studies mentioned above, this aberrant activation pattern can be detrimental to individuals with aMCI and be responsible for their episodic memory dysfunction.

The angular gyrus has been reported consistent activations in a variety of tasks (see Seghier, 2013 for review), particularly during successful episodic memory retrieval (e.g., Vilberg and Rugg, 2008; Spaniol et al., 2009). As reviewed by Rugg and Vilberg (2013), evidence from resting state connectivity and DTI tractography (Uddin et al., 2010; Sestieri et al., 2011), especially the findings that the performance of recollection-based recognition, is associated with enhanced connectivity between the angular gyrus and hippocampus (McCormick et al., 2010), indicating that the angular gyrus may play an important role in the memory network despite many different theories (e.g., bottom-up attentional re-orienting, episodic buffer, episodic convergence zone). In the present study, however, individuals with aMCI demonstrated lower activation likelihood in this region not during retrieval but the encoding stage. As a part of the default mood network, some studies reported that the deactivation of angular gyrus during encoding is beneficial for the memory performance (Daselaar et al., 2009; Uncapher and Wagner, 2009). Nevertheless, there are also opposite findings suggesting that the left angular gyrus activity is greater during successful vs. unsuccessful episodic encoding (Maillet and Rajah, 2014). Elman and colleagues demonstrated the dynamic changes in angular gyrus during encoding. The angular gyrus activity decreased when the stimulus initially presented and increased during an elaborative representational encoding process (Elman et al., 2013). The lower activation likelihood of left angular gyrus during episodic encoding in aMCI individual in the present study indicated the deficit of this representational process which results in their memory impairment. Alternatively, this lower activation likelihood during encoding may be a compensative inhibition because of its role of default mood network. Due to the ALE analysis technique, the present study cannot fully prove which explanaiton is more reasonable. From the tasks perspective (intentional encoding) used in the studies which provided the foci (i.e., Machulda et al., 2009; Hampstead et al., 2011), the first hypothesis is more plausible.

### Other regions

Relative to healthy controls, individual with aMCI demonstrated lower activation likelihood in the anterior portion of left superior temporal gyrus (BA 38) during encoding. The lateral temporal lobes are not the key structures for episodic encoding and retrieval processes, but these regions are reported to be important for semantic knowledge representation. Particularly, the portions in anterior temporal lobes (BA 38) have been suggested as “hubs” which converge the distributed attributes to a common set of semantic representations, regardless of the task (see Patterson et al., 2007 for a review). Recent studies showed the interaction between the episodic memory and semantic memory network during lexicalization (similar with a lexical episodic memory task) with the superior temporal gyrus involved in the novel words memory (Takashima et al., 2014). For AD patients, the superior temporal gyrus is among one of the first areas affected by the disease (Ding et al., 2009), and it had been found that the activity in this portion during memory encoding predicted better performance on measures of cognitive status across AD patients (Diamond et al., 2007). Thus, the deactivation of superior temporal gyrus in aMCI individuals in the present study may reflect impairment of semantic knowledge processing during episodic encoding. This is in line with studies demonstrating that this region is related to the semantic deficit in MCI participants (Vandenbulcke et al., 2007; Clark et al., 2014).

In encoding conditions, portions of the posterior cingulate cortex (PCC) showed less likelihood of activity among aMCI individuals than controls in the present study. As a part of the memory retrieval network, the PCC involves in elaborative retrieval and evaluation of self-referential information (Shannon and Buckner, 2004; Wheeler and Buckner, 2004; Rugg and Vilberg, 2013). Several studies have observed the dysfunctional lower activity in PCC in MCI during episodic retrieval (Johnson et al., 2006; Ries et al., 2006); the deficit of PCC during encoding in MCI, however, was rarely reported. An ALE meta-analysis reported that the PCC was significantly less activated during encoding in early AD patients than controls (Schwindt and Black, 2009). As a transitional stage, individuals with MCI have volumetric and metabolic decline in PCC (Nestor et al., 2003), and the level of metabolism and regional blood flow in PCC were able to predict the conversion to AD (Chételat et al., 2003). Therefore, the less activation likelihood among aMCI individuals in PCC during encoding may reflect the dysfunction in the representational encoding process and result in their following elaborative retrieval.

Individuals with aMCI also showed lower activation likelihood in the left fusiform gyrus, bilateial cuneus, left putamen, and right thalamus, as well as elevated activation likelihood in the right lateral globus pallidus and a portion of left thalamus during encoding. These results were similar as the situation in AD patients (Schwindt and Black, 2009). These regions are usually not reported as key nodes involving in either episodic encoding or retrieval mode. However, a successful episodic encoding is subserved by more widespread cortical regions, not only the key notes (MTL, PFC, areas of posterior parietal), but also the more fundamental structures such as the thalamus, fusiform gyrus, and cuneus (Patterson et al., 2007; Akanuma et al., 2009). These differences may prove more basic task-specific processing or the pathological dysfunction observed in individuals with aMCI.

### A widespread episodic memory network impairment

As reviewed by Shimamura (2014), PFC as an executive-control system, selects and updates information of sensory, conceptual, and emotional features that constitute an episodic memory. Then, the MTL binds the features as an encapsulated memory to make each item of episodic memory information distinct or separable from others. For retrieval, neuroimaging studies have shown a general network, including the MTL structure, retrosplenial/posterior cingulate, ventral posterior parietal cortex (vPPC), and mPFC (Rugg and Vilberg, 2013). Retrieval typically starts within PFC which facilitates the search through memory and activates pertinent event features. The MTL functions by activating event features through relational bindings (Shimamura, 2014). Because of their connections with the hippocampus and parahippocampal crotex, the PCC and mPFC may play a role in the processing of contextual information (Kveraga et al., 2011; Aggleton, 2012). The ventral posterior parietal cortex centered on the angular gyrus is also a part of the retrieval network due to its interconnection with the MTL and posterior cingulate cortex (Uddin et al., 2010; Sestieri et al., 2011), although its exact role has not been confirmed (see review Rugg and Vilberg, 2013; Shimamura, 2014).

The present study indicated a broad damaged network of episodic memory in aMCI individuals, which involves all the core structures in encoding, retrieval, and some more basic brain structures. Although the additional activations both during encoding and retrieval in the dorsal frontal cortex could refer to a form of compensation mechanism, this is still not a normal situation in contrast to healthy older adults. The original hypothesis which attributed the memory impairment in aMCI specifically to degeneration of the MTL structure (Petersen et al., 2001) is now viewed as incomplete: functional brain imaging revealed hypometabolism not only in the bilateral MTL, but also in the PFC, angular gyrus, precunes, PCC, and several more basic structures such as the thalamus, fusiform gyrus, and cuneus, which apparently constitute a complicated network that is crucial for the formation and representation of new memories.

In addition, as indicated by several studies, the connectivity between the key nodes of the mnemonic network (such as PFC, MTL, posterior parietal cortex) is very important to memory process (Ranganath et al., 2005). This connectivity even plays a significant role in compensating for reduced regional activity during successful memory processing in aging. For instance, Oh and Jagust (2013) reported that cognitively normal older adults without β-amyloid deposition (a prominent feature of Alzheimer's disease associated with neural alterations and episodic memory decline) showed a reduced regional brain activation with increased task-related connectivity (compared with young adults) between parahippocampal gyrus and prefrontal cortex, and the degree of connectivity was related to memory performance. However, cognitively normal older adults with β-amyloid deposition showed no such increased task-related network connectivity. Due to the limitations of the ALE technique, the present study is unfortunately only able to describe the differences of brain activation between aMCI individuals and healthy controls. Recent studies have proved that the functional connectivity within this mnemonic network is declined in individuals with aMCI during resting state, which is associated with their memory impairment (Li et al., 2013; Dunn et al., 2014). The future research probably requires the investigation of the functional connectivity characters within this mnemonic network in individuals with aMCI during episodic encoding and retrieval processing.

### Limitations

Firstly, due to the specificity of our research objective and technique, we were forced to ignore a number of factors that varied across the included papers. The limitations of the present meta-analysis were largely related to the variety of task paradigms related to episodic memory across the included studies (as shown in Table 1), such as the stimuli, baseline contrast, paradigm design (i.e., block vs. event-related), or statistical method (i.e., univariate vs. multivariate). As mentioned in the similar ALE meta-analysis (Schwindt and Black, 2009; Browndyke et al., 2013), we were unable to control the potential confound of effort and task difficulty between groups across studies due to the technique.

Secondly, participant characteristics such as age, gender, and disease severity were other uncontrollable factors. Especially the subtypes and severity of the cognitive impairment in aMCI individuals across the papers were important issues and could have a significant impact on the pattern of activation. Although, only the studies with aMCI participants were included in the present study, it was hardly impossible to control the single and multiple dysfunctions or the severity of impairment in some cognitive functions. The heterogeneous nature of the individuals with aMCI leads us to be cautious with our interpretations, for the activation in various brain regions may be a time of dynamic change between increases and decreases with the cognitive impairment progress in individuals with aMCI (Celone et al., 2006; Clément and Belleville, 2010). As reviewed by Gainotti et al. (2014), the spread of the neurofibrillary tangles from the subcortical noradrenergic structures to the perirhinal/entorhinal cortices and to the hippocampus may be the substrate of the sequence of semantic and episodic memory disorders spanning from the early subclinical to the aMCI stage, and other cognitive defects and AD become apparent when it spreads to the neocortical associative areas. Unfortunately, due to the limited literatures, it was impossible to divide the aMCI individuals into several subtypes according to the severity of their cognitive impairment.

Thirdly, a meta-analysis study revealed that individuals with MCI affected structurally in the (trans-) entorhinal and hippocampal regions (Schroeter et al., 2009), however, majority of the studies included in the presents analysis did not account for brain atrophy in interpreting activation differences, except several such as (Lenzi et al., 2011).

## Conclusion

Despite a number of challenges inherent in functional imaging of the individuals with aMCI, the present ALE meta-analysis encouragingly reveals that certain findings are consistent across the episodic memory literature and laboratories. Individuals with aMCI definitively demonstrated an abnormal pattern in a widespread episodic memory network, not only in the bilateral MTL, but also in the PFC, angular gyrus, precunes, PCC, and even some more basic structures such as the thalamus, fusiform gyrus, and cuneus. The results of current ALE meta-analysis further support that the abnormal condition in the functional brain network of aMCI individuals may not be “mild” at all, but even more severe in nature.

## Author contributions

PW coded, analyzed and interpreted data, drafted the manuscript. JL conceived the idea, designed the study, and participated in writing up and revising the manuscript. LH, HL, and RL assisted coding and data analysis. All authors reviewed the manuscript.

## Funding

This research was supported by the National Natural Science Foundation of China (31271108, 30911120494, 31070916, 31671157, and 31400895); the National Science and Technology Pillar Program of China (2009BAI77B03); the Knowledge Innovation Project of the Chinese Academy of Sciences (KSCX2-EW-J-8), CAS/SAFEA International Partnership Program for Creative Research Team (Y2CX131003), the Institute of psychology, Chinese Academy of Sciences (111000C038, Y3CX151005), the Pioneer Initiative of the Chinese Academy of Sciences, Feature Institutes Program, TSS-2015-06, and CAS Key Laboratory of Mental Health, Institute of Psychology (KLMH2014ZK02, KLMH2014ZG10).

### Conflict of interest statement

The authors declare that the research was conducted in the absence of any commercial or financial relationships that could be construed as a potential conflict of interest.
