# Supplementary material for: Mild Cognitive Impairment Is Not “Mild” at All in Altered Activation of Episodic Memory Brain Networks: Evidence from ALE Meta-Analysis
Source: Front Aging Neurosci. 2016 Nov 7;8:260. doi: 10.3389/fnagi.2016.00260 (PMC5097923; doi:10.3389/fnagi.2016.00260)
Supplement: Supplementary file 1 [file Table1.PDF]

Supplemental table 1

Results of ALE analyses for each group.

Region (Left/Right, Brodmann area)	X	Y	Z	Cluster size (mm^3)	ALE ($\times 10^{-2}$)
Encoding					
NC					
Frontal Lobe					
Inferior frontal gyrus (L, 9)	-42	10	30	2112	2.84
Precentral gyrus (L, 6)	-44	0	38		1.49
Medial frontal gyrus (L, 6)	-10	2	54	456	2.00
Middle frontal gyrus (R, 46)	44	30	20	280	1.55
Precentral gyrus (R, 9)	36	16	30	168	1.41
Superior frontal gyrus (L, 6)	-2	20	50	160	1.47
Limbic lobe					
Parahippocampal gyrus (R, 27)	22	-30	-6	2000	2.01
medial globus pallidus (R)	16	-8	-10		1.90
Parahippocampal gyrus (L, 27)	-20	-30	-4	152	1.16
Sub-lobar thalamus (L)	-16	-32	2		1.28
Parahippocampal gyrus (L, 36)	-28	-36	-12	120	1.37
Parietal lobe					
Precuneus (L, 19)	-28	-70	36	848	2.16
Precuneus (R, 7)	20	-64	36	552	1.94
Temporal lobe					
Fusiform gyrus (L, 37)	-32	-50	-14	520	1.71
Occipital lobe					
Cuneus (R, 17)	26	-80	18	248	1.50
Lingual gyrus (R, 18)	18	-88	-4	160	1.43
Middle occipital gyrus (L, 19)	-28	-80	16	104	1.39
Sub-lobar					
Thalamus (L)	-10	-12	10	216	1.50
Insula (L, 13)	-34	24	2	136	1.46
Amygdala (L)	-20	-10	-12	112	1.29
Cerebellum					
Anterior lobe culmen(R)	24	-52	-10	448	1.84
Posterior lobe declive (L)	-38	-70	-12	112	1.37
MCI					
Frontal lobe					
Precentral gyrus (L, 6)	-42	0	36	1632	2.84
Middle frontal gyrus (R, 9)	46	28	24	416	2.27
Medial frontal gyrus (L, 6)	-6	6	50	312	2.01
Precentral gyrus (R, 6)	42	2	40	128	1.56
Limbic lobe					

Sub-lobar thalamus pulvinar (L)	-16	-28	2	720	1.76
Parahippocampal gyrus (L, 36)	-28	-36	-12		1.60
Parahippocampal gyrus (L, 27)	-22	-32	-4		1.47
Parahippocampal gyrus (R, 28)	22	-28	-8	440	1.80
Parahippocampal gyrus (R, 36)	28	-36	-14	112	1.45
Parietal lobe					
Precuneus (L, 7)	-28	-68	40	1048	2.67
Precuneus (R, 7)	22	-60	36	224	1.73
Superior parietal lobule (R, 7)	32	-64	50	128	1.55
Temporal lobe					
Fusiform gyrus (L, 37)	-40	-52	-12	160	1.68
Occipital lobe					
Lingual gyrus (L, 18)	-16	-90	-10	1264	0.04
Lingual gyrus (R, 18)	18	-90	-2	1248	2.59
Middle occipital gyrus (R, 18)	28	-88	0		2.31
Middle occipital gyrus (L, 19)	-30	-80	16	280	1.78
Cuneus (R, 17)	26	-80	18	136	1.54
Cerebellum					
Anterior lobe culmen (R)	24	-52	-12	256	1.76
Retrieval					
NC					
Frontal lobe					
Superior frontal gyrus (R, 6)	6	15	50	432	1.36
Parietal lobe					
Precuneus (L, 7)	-30	-66	40	480	1.46
Temporal lobe					
Middle temporal gyrus (L, 39)	-54	-62	16	136	0.89
Occipital lobe					
Cuneus (R, 18)	4	-76	30	240	0.98
Sub-lobar					
Extra-nuclear (R, 47)	38	22	-8	288	1.11
Cerebellum					
Posterior lobe, declive (L)	-30	-74	-18	480	1.46
MCI					
Frontal lobe					
Medial frontal gyrus (R, 6)	6	22	42	568	2.56
Parietal lobe					
Precuneus (R, 7)	32	-66	42	208	1.70
Precuneus (L, 7)	-28	-64	44	136	1.63
Occipital lobe					
Lingual gyrus (R, 18)	18	-90	-4	648	2.90
Sub-lobar					

Claustrum (R)	32	26	-2	512	2.33
Cerebellum					
Anterior lobe, dentate (R)	20	-54	-21	160	1.66

Notes. ALE = activation likelihood estimation. Coordinates in stereotactic space of MNI.
